# Experiences of nursing students during their mental health clinical training at a general hospital in Namibia

**DOI:** 10.4102/curationis.v46i1.2441

**Published:** 2023-08-25

**Authors:** Daniel O. Ashipala, Daniel Mbishi, Louise Pretorius

**Affiliations:** 1 Department of General Nursing Sciences, School of Nursing and Public Health, Faculty of Health Sciences and Veterinary Medicine, University of Namibia, Rundu, Namibia

**Keywords:** experiences, mental health, clinical training, nursing students, general hospital

## Abstract

**Background:**

Clinical training is important because it constitutes more than half of the formal courses in nursing education. Accordingly, it is important for institutions of higher learning to continually explore the experiences of nursing students during their clinical placement. These experiences can be used to promote a positive clinical learning environment for students. However, the experiences of nursing students during their mental health clinical training in Namibia have not been extensively researched.

**Objectives:**

The aim of this study was to explore and describe the experiences of nursing students during their mental health clinical training at a general hospital in Namibia.

**Method:**

A qualitative, exploratory, descriptive and contextual research design was followed as the basis for conducting the study. A sample of 15 nursing students was conveniently selected from the target population of 73 nursing students. This sample size was determined by the saturation of data as reflected in repeating themes.

**Results:**

The following three themes emerged: collating theory and mental health practice experiences, facing challenges in clinical placements, and recommendations to ensure effective learning about mental health.

**Conclusion:**

The use of a general hospital for clinical placements within an undergraduate nurse training course led to some concerns regarding the relevance and appropriateness of such experience within a nursing programme.

**Contribution:**

The findings have important implications for the training of undergraduate nursing students in general hospitals. It can be utilised to develop strategies to improve positive clinical practice placement and develop clinical skills for undergraduate nursing students in general hospitals.

## Introduction

Hospital wards have been the traditional clinical training setting for nursing students. Depending on the country and the healthcare system, students also receive clinical training in community care, mental healthcare, nursing homes, as well as in non-traditional settings such as parishes, prisons, or children’s day care (Harwood et al. 2009). Clinical training for nursing students is important in many respects of their nursing programme. In Namibia and in many other countries, clinical education forms more than half of the formal courses in nursing. Therefore, clinical education is an essential and integral part of nursing education programmes (Aghamohammadi-Kalkhoran et al. 2011). Because nursing is a performance-based profession, clinical learning environments play an important role in the acquisition of professional abilities and clinical skills to prepare nursing students to enter the nursing profession (Jonsén, Melender & Hilli 2013).

According to Newton et al. (2010), clinical placement is regarded as the setting where the skills, knowledge and attitudes that are developed in the theoretical part of the curriculum are applied, developed and integrated (Newton et al. 2010). Moreover, Jamshidi et al. (2016) describe the clinical area of nursing education as of great importance for nursing students in their decision to pursue nursing as a profession.

In the mental health setting of Australia, nursing students traditionally undertook a 4-week block for clinical training (Boardman et al. 2018). Under the block (or rotational) model, students are ‘buddied’ with a registered nurse for each shift, while a clinical supervisor serves as the primary instructor for a small group of students across different wards or units. However, in Norway, an integrated clinical learning model, where preceptors mentor students on an individual basis, has been used successfully in the clinical learning environment (Kirkbakk-Fjær, Andfossen & Hedelin 2015). This flexible model provides students with the opportunity to work morning, afternoon, night and weekend shifts during the clinical block. Accordingly, there is a need to improve the evidence base for such a flexible model to be applied in mental health clinical training. In the context of this study, a fixed rotational model of 2 weeks per placement is used which is similar to the integrated model of Norway. Clinical preceptors and instructors are following the students on an individual basis to ensure an efficient placement. This also applied to the mental health placement.

Studies have pointed to the fact that there is a need for improved mental healthcare in Namibia (Ashipala, Wilkinson & Van Dyk 2016). Mental healthcare often receives low priority, is often seen as secondary to physical healthcare, and is subsequently relegated to the fringes of care and planning. Facilities for mental healthcare lag far behind other areas of healthcare and for the majority who live in rural areas of Namibia, there is no mental healthcare at all, according to the Ministry of Health and Social Services (MoHSS 2005). This includes a lack of appropriate infrastructure and a lack of knowledgeable and skilled healthcare professionals able to manage patients with mental disorders; in addition, the inability of those healthcare professionals also lies in the transfer of knowledge and skills to nursing students placed for clinical practice.

Namibia has two mental health referral hospitals, namely, the Windhoek Mental Health Care Centre and the Oshakati Psychiatric Unit located inside the Oshakati Intermediate Hospital (a specialised hospital). However, in recent years, another general hospital in the north-east of Namibia, within the context of this study, has recorded an increase in patients diagnosed with mental disorders (MoHSS 2005). Mentally ill patients requiring admission are involuntarily admitted against their own will as either one of their family members, friends or someone declared them mentally sick, in accordance with mental health act section 4 of *Mental Health Act 18 of 1973 (Act No 18 of 1973)* has to be signed on admission. A total of 8017 mental health patients were admitted to the latter in 2020. This high number of mental health cases led to a decision by the management of the School of Nursing and Public Health (SoNPH) to allocate final-year students in the above-mentioned hospital for mental health clinical training as opposed to a placement in Oshakati Intermediate Hospital and Windhoek Mental Health care Centre, which would be cost-intensive for the students.

Although this general hospital is used as a clinical allocation facility for nursing students, it does not have a specialised mental health unit. Mentally ill inpatients are admitted within the two medical wards to which the students were allocated for their mental health practice due to the absence of any other facility as alluded to above. The ward had only one mental health specialised trained nurse, who was also responsible for all other patients in the medical ward. The limited number of mental health trained nurses in the unit contributes to the limited transfer of skills and knowledge to students (MoHSS 2013a) placed for clinical placement. The nurses in the ward consider themselves inadequately equipped to provide effective clinical teaching and supervision for the nursing students allocated for mental health clinical training (MoHSS 2020).

There is limited international evidence on experiences of nursing students in a mental health setting, let alone mental health in a general ward setting. The aim of this study was to explore and describe the experiences of nursing students during their mental health clinical training at a general hospital in the north-east of Namibia. This will inform future targeted placement strategies to successful development of relevant clinical skills, and strongly influence future career choices in mental health which are seen to have a significant impact on students’ attitudes towards mental health as an area of specialisation. It will further inform the SoNPH management as to whether the decision to place students in such general setting for mental health placement, as informed by statistics, was indeed in the best interest of the students.

## Research methods

### Research design

A qualitative, exploratory, descriptive and contextual research design was used as a basis for this research conducted in an urban-based general hospital in the north east of Namibia. According to Maree (2016), a qualitative research design is naturalistic, focusing on natural settings where interactions occur. Exploratory research is one which aims at providing insights into and an understanding of the problem faced by the researcher (Polit & Beck 2017). Descriptive research is one which aims to describe a phenomenon and its characteristics (De Vos et al. 2017). Therefore, it was a suitable design for exploring and describing the experiences of nursing students during their mental health clinical placement at a general hospital in the north-east of Namibia. The current study is reported according to the Standards for Reporting Qualitative Research (SRQR) (O’Brien et al. 2014) and comprises analysis of open-ended responses where participants were asked to explore and describe the experiences of nursing students during their mental health clinical training at a general hospital in the north-east of Namibia.

### Study setting

The study was conducted at a general hospital situated in the north-east of Namibia for the reason already alluded to above. For their mental health clinical placement, students are placed in the outpatient department and the male and female wards of this hospital for a period of 2 weeks. The bed capacity for patients with a mental illness, admitted to this hospital is less than 10. Four beds in the male ward and four in female ward can be increased to house 10 inpatients in times of desperate need; this amid reports that this town is one with the most patients with mental illness in Namibia (MoHSS 2020). The hospital also has an Outpatient Department which sometimes serves up to 100 mental health patients a day (MoHSS 2013b). Outpatients come to this facility mainly for follow-up.

### Population and sampling

Participants in this study were fourth-year undergraduate nursing students who were enrolled for the Bachelor of Nursing Science (Clinical) (Honours) programme at the University of Namibia Rundu campus and who were placed for their 2-week clinical training for mental health at this general hospital in Namibia. This study focused on the undergraduate student nurses registered for the Bachelor of Nursing Science (Clinical) (Honours). The SoNPH at the satellite campus was established in 2017 and offers one nursing programme only, namely, a Bachelor of Nursing Science (Clinical) (Honours). The higher education institution is deemed as the main Higher Education Institution (HEI) involved in the training of nurses to become professional nurses and were using the 2-week block system at the time of this study (Shatimwene, Ashipala & Kamenye 2020). The mental health module is introduced in the fourth year of the degree programme. Students have about 160 practical hours for the 4-week allocation in mental health. This clinical exposure continues over 28 weeks in a mental health unit, to accommodate the large number of students in the fourth year. During their clinical placement in a mental health unit, students are issued with a clinical register that requires completion by the end of this period. This register will account for admission to the examination. The aim of this clinical placement is to enable students to correlate the theoretical knowledge acquired in classroom with what they observe and hear in the clinical setting and in their daily lives.

In the 2020 academic year, 73 fourth-year nursing students were registered in this programme at the School of Nursing, Rundu campus of the University of Namibia. This constituted the population for this study. For this study, a convenient sampling technique was used. Convenient sampling allows an equal opportunity of participants to be part of the study (Grove, Gray & Burns 2017). The inclusion criteria in this study were: fourth year undergraduate nursing students registered for the Bachelor of Nursing Science (Clinical) (Honours) who were willing to participate and consented to the study. The number of students who were interviewed was determined by data saturation, as reflected in repeated themes. Data saturation was reached after 15 interviews.

### Data collection methods

In this study, data were collected by the researcher using semi-structured individual interviews with participants who met the inclusion criteria. To be a participant in this study, participants have to meet the following criteria: (1) should be a fourth-year nursing student registered for mental health nursing, (2) willing to participate in the study by signing an informed consent and (3) available at the time of data collection.

After initial contact with the participants selected according to the sampling criteria for this research, informed consent was obtained to tape record the interviews. The date, time and place of the interview were duly confirmed. The interview session lasted for approximately 45 min – 50 min. During the interview, the researcher took field notes and used follow-up questions to probe for more detailed exploration. Prior to data analysis, the field notes were combined with the transcribed interviews to provide insight into the situation during the interview. The main question posed during the interviews included the following: *What were your experiences during the mental health clinical training in the hospital?*

### Data analysis

In this study, thematic analysis was used to analyse the data. This is deemed to be the most reliable method used in qualitative research as it is fairly systematic and allows the researcher to organise the information into themes and sub-themes (Polit & Beck 2017). Firstly, the researchers familiarised himself with all the research data by reading and re-reading all the interview transcripts before making data notes. Secondly, the researcher developed initial data codes, which informed themes and categories from the data. Moreover, the researcher identified predominant themes and categories from a list of various codes which needed to be reduced to form significant themes and categories pertinent to the data. Furthermore, he reviewed and refined all themes and categories, through eliminating themes that, after some consideration, were removed due to a lack of evidence from data. Main themes and sub themes were finalised and named, with the researcher getting an overall understanding of what each term contained before writing up the research findings. The independent co-coder and the researcher subsequently underlined the units of meaning that related to the identified categories. These units of meanings were then placed into the major categories and sub-categories that were identified. The independent coder was requested to also analyse the data according to thematic analysis method, independently of the researcher. The two analyses were then compared to ensure trustworthiness. The independent coder was selected because of a Doctorate in Nursing education, where a qualitative interview method was utilised. Phrases that pertained directly to the phenomenon and captured the essence of the experience were extracted as a unit of general meaning. The categories or units of meaning were clustered and those redundant were discarded. Subsequently, three themes emerged.

### Measures of ensuring trustworthiness

The trustworthiness of the study was ensured by using the criteria of Lincoln and Guba (1985), namely credibility, dependability, transferability and confirmability. Credibility in this study was achieved through prolonged engagement with, and persistent observation of, the participants. The researcher remained in the field for 6 weeks throughout the interview process in order to gain an in-depth understanding of the phenomenon. A tape recorder was used during the interviews, with those recordings being transcribed and the recordings and transcripts being compared to confirm that they were from the same participants. Transferability was achieved through a complete description of the research design and methodology used, as well as through the convenient selection of participants. The data collected were described as accurately as possible; full or thick descriptions of the experiences of the participants can be made available upon enquiry. Dependability was achieved through the use of audio recordings and transcripts can be made available upon enquiry. The data collected are also supported with related literature and the data collection methods are comprehensively described. In order to confirm the data, a consensus meeting with an independent coder, who was experienced in qualitative research and held a doctorate in nursing education, was utilised. Additionally, verbatim transcription of the interviews and re-reading of these and the field notes enabled the researcher to get a better understanding of what the participants said about their experiences of nursing students during their mental health clinical training at a general hospital in the north-east of Namibia.

### Ethical considerations

Approval for this study was granted by the University of Namibia’s School of Nursing Research Ethics and Review Committee (SoNREC) (reference no. 49/2020) and the MoHSS Research Committee (reference no. DM/2020). Informed consent was sought verbally from the participating respondents and through their signing of a consent form. Participation in the study was purely voluntary, and the interviewees were free to withdraw at any time, although this was not encouraged. The participants were also assured of anonymity, with the use of pseudonyms on the research tools instead of names, as well as confidentiality. The data were stored safely and will be disposed of according to the university’s policies.

## Results

### Socio-demographic description of study participants

A total of 15 participants were interviewed. Participants were all fourth-year nursing students completing their Bachelor of Nursing Science degree programme at the University of Namibia. Of the 15 respondents, eight were male while seven were female with an age range of between 20 and 40 years. The three themes that emerged from the data analysis are indicated in Table 1.

**TABLE 1:** Themes and sub-themes that emerged from the data analysis.

Themes	Sub-themes
1. Collating theory and mental health practice experiences	1.1 Apply what was taught in theory 1.2 Real-life exposure to mental health builds experience
2. Challenges in clinical placements	2.1 Absence of appropriate mental health units 2.2 Lack of trained psychiatric nurses 2.3 Insufficient exposure to mental health patients
3. Recommendations to ensure effective clinical learning about mental health	3.1 Student placements at appropriately equipped hospitals 3.2 Time allocation to be increased for more exposure 3.3 Building mental health institutions to accommodate increasing numbers 3.4 Health Ministry to appoint more nurses and doctors specialising in mental health at institutions

### Theme 1: Collating theory and mental health practice experiences

This theme encapsulates the participants’ experiences as nursing students with regard to their mental health placement at the general hospital. Experience is the process by which conscious beings perceive the world around them. In this case, experiences may include active awareness on the part of the experienced student. The students referred to two experiences that they had undergone. The sub-themes of this theme include: apply what was taught in theory and real-life exposure to mental health builds experience.

#### Sub-theme 1.1: Apply what was taught in theory

This sub-theme describes the way the students expressed their experiences in the general hospital mental health wards as a learning opportunity for them. The participants stated that:

‘It was good in the sense we got an opportunity to apply what we were taught in theory and also get an opportunity to meet real patients and the diff erent conditions of patients and the way they are behaving.’ (P2, female, 23 years old)

‘I personally had a good experience because I got the opportunity to link what we were taught in theory to practice. Most of the conditions that we were taught in theory I was able to see them and I assessed them.’ (P3, male, 25 years old)

#### Sub-theme 1.2: Real-life exposure to mental health builds experience

The participants revealed how their placement in the mental unit and exposure to mental illness would be of benefit to them one day. The participants narrated that:

‘It was a good experience, educative; we got to learn how to evaluate mental patients. The exposure to mental health unit at the hospital, assessing and evaluating mentally ill patients, boosted my confidence to work in the mental unit in the future.’ (P5, female, 24 years old)

‘I was exposed to different types of mental conditions. I was able to make a fair comparison between the practical and the theoretical part in mental health issues in Namibia and common elsewhere in the world.’ (P4, male, 26 years old)

### Theme 2: Challenges faced in clinical placements

This theme entails the participants’ descriptions of the challenges that they faced and how they affected their clinical learning experience as students. Mental health clinical training is an important part of nursing education and students’ exposure to the mental health environment is one of the most important factors influencing the teaching-learning process in nursing settings. Accordingly, identifying the challenges of nursing students in the mental health learning environment will enhance the training and improve its planning and the quality of student development. The sub-themes derived from this theme were identified as follows: absence of appropriate mental health units, a lack of trained psychiatric nurses and insufficient exposure to mental health patients.

#### Sub-theme 2.1: Absence of an appropriate mental health unit

Some participants in this study described the set-up of the hospital as not ideal to meet the clinical learning outcome in mental health, as it has only one room for all the patients with mental illness. Therefore, it is not conducive for learning or for patient care. Participants mentioned that:

‘There is no ward to put mental health patients, and these patients need a special ward to take care of them and cater for their needs. So, we didn’t get the opportunity to see how a proper mental health unit was supposed to look like.’ (P8, male, 24 years old)

The students lamented that the units where patients with mental illness are kept is very small. As a result, the environment is not beneficial for either the students or the patients.

‘The facility seclusion rooms are not really up to standard to incorporate people that are able to kill themselves or things like that. They are kept in one room, even those ones that are not aggressive and that are not good. This practice is also not good for our learning experience.’ (P12, female, 27 years old)

#### Sub-theme 2.2: Lack of trained psychiatric nurses

This sub-theme reports on the way in which the lack of mental healthcare nurses affected the students during their mental health clinical training at this hospital. Participants in this study mentioned that their clinical learning as student nurses was inadequate and their role in providing mental healthcare was underdeveloped. Appropriately trained nurses can contribute to the promotion of mental health and the prevention and treatment of mental disorders. The participants lamented that:

‘The other thing is also that there are no nurses who are specialised in psychiatry that were able to help us, hence most of the time it was just our lecturer who will come to assist us.’ (P15, female, 26 years old)

‘At this hospital, there are two units where we keep mental health patients but each one of those has one room and there is only one mental nurse in the whole hospital and we didn’t really get enough support.’ (P14, male, 24 years old)

#### Sub-theme 2.3: Insufficient exposure to mental health patients

The participants also explained that they did not spend enough time in the mental health unit. This sub-theme illustrates how limited time influences students’ clinical learning at this hospital and how it posed a challenge to their pursuit of practical knowledge and learning objectives. Participants indicated that:

‘For me I had a few challenges, the first one is the time frame that students are exposed to the mental health unit was too short.’ (P6, female, 26 years old)

‘Another challenge is that we were not able to carry out procedure from our practical book like the occupational therapy due to lack of time.’ (P7, male, 27 years old)

### Theme 3: Recommendations to ensure effective learning about mental health

This theme emerged from the participants’ responses when they were asked what could be done to meet students’ needs to ensure effective learning in mental health. The sub-themes in this theme include student placements at appropriately equipped hospitals, time allocation to be increased for more exposure, mental health institutions to be built to accommodate increasing numbers, and the MoHSS to appoint more nurses and doctors specialising in mental health at institutions.

#### Sub-theme 3.1: Student placements at appropriately equipped hospitals

Some participants suggested that students should be placed at appropriately equipped hospitals so that they could be exposed to a real mental health unit. Participants in this study described a well-equipped hospital with adequate doctors, nurses, and all medical equipped, the hospital helps to provide treatment to a person from mental illness. The participants stated that:

‘It’s best if they send students to hospitals are best equipped to help students learn about mental health.’ (P8, male, 24 years old)

‘In the following academic year if things go back to normal, students must go to different mental health unit where set are appropriate.’ (P9, female, 27 years old)

#### Sub-theme 3.2: Time allocation to be increased for more exposure

The participants recommended that the time frame for the mental health placement at the hospital should be increased so that the students could be exposed to mental health patients for a longer period. The participants suggested that:

‘Regarding time allocation to conduct our practical’s in the mental healthcare should be increased to from the current two weeks to a one-month block.’ (P11, female, 22 years old)

‘For me I had a few challenges, the first one is the time frame that students are exposed to the mental health unit was too short and there are few nurses to assist the students in the mental health unit. In the end i could not complete my practical register on time.’ (P15, female, 25 years old)

‘We were placed within two weeks and the content was a lot to be covered during just two weeks. I suggested maybe I would suggest maybe the management of UNAM [*University of Namibia*] maybe could increase the time frame of student being exposed to mental health unit.’ (P16, male, 27 years old)

#### Sub-theme 3.3: Building mental health institutions to accommodate increasing numbers

The participants suggested that the government should build more specialised mental health institutions that could accommodate the rapidly increasing numbers of mentally ill patients in the country. The participants recommended that:

‘The government must build more mental health institution to accommodate more patients.’ (P13, male, 23 years old)

‘The current structure where mental ill patients are cared for together with other patients in a general ward is not ideal for learning. The ministry should at least explore the possibility of building a specialised mental health unit or redesign and improvise the current structure to make it a bit suitable for learning considering that this is a training hospital.’ (P17, male, 25 years old)

#### Sub-theme 3.4: Ministry of Health and Social Services to appoint more nurses and doctors specialising in mental health at institutions

The participants recommended that the Health Ministry should hire doctors and nurses who are trained in psychiatry. They recommended that:

‘They should encourage nurses to specialise in mental health or they can recruit nurses that have specialised in mental health.’ (P1, male, 29 years old)

‘The Ministry of Health should consider sending nurses on staff development to pursue postgraduate qualification in mental health even in South Africa.’ (P10, male, 27 years old)

‘The Health Ministry must employ more nurses that have specialty in mental health.’ (P14, male, 24 years old)

## Discussion

Participants in this study reported that they had a good learning experience during their mental health clinical placement, mentioning that they had learnt a lot about mental health and how to manage a mental health patient.

Additionally, the study found that participants in this study had the opportunity to put into practice what they had learnt in theory and to make skilled observations when caring for patients with mental illness. These findings are similar to that of Peters, McInnes and Halcomb (2015), who reported that clinical placements are an innovative strategy for high quality teaching and learning experiences among undergraduate nursing students. Similarly, a study conducted by Ngu (2021) at a university in the Cape Town reported that the integration of theory and clinical practice is of utmost importance in an undergraduate degree programme.

Clinical placements in mental health settings are known to improve nursing students’ mental health nursing skills and knowledge of mental illnesses (Patterson et al. 2016). However, in this study participants reported that during their placement in mental health facilities they learnt how to evaluate mental patients. Thus, it is imperative to provide nursing students with placement opportunities in a mental health unit that will help them improve their learning and develop appropriate professional skills, care and attitudes. These findings agree with the work of Stuhlmiller and Tolchard (2019) who argued that, offering community opportunities along with exposure to positive instructor beliefs about mental illness, can both improve student attitudes prior to the completion of their nursing studies and may encourage entry into mental health as a nursing option post-education. The fact that exposure to mental health is a good learning experience for the students is supported by Cowley et al. (2016), who reported that mental health education exposure can improve students’ clinical confidence in practising with people with a lived experience of mental illness and can prepare nursing students for professional practice. A literature review conducted by Happell et al. (2015) about clinical placements in mental health similarly revealed that clinical placements of nursing students in mental health facilities improve students’ skills, knowledge, attitudes towards people with mental health issues and confidence, as well as reducing their fears and anxieties about working in mental health. A study of Lim et al. (2020) also highlighted the need to improve current mental health clinical placements to better build nursing students’ confidence in caring for mentally ill patients.

One of the challenges that the study found was the absence of an appropriate mental health unit at the general hospital. Participants reported that seclusion rooms in the mental health unit were not well equipped in order to incorporate people that are able to kill themselves and all the mentally ill patients were placed in one room. Owing to the lack of ideal facilities and rooms at the hospital, the students did not get to see what a proper mental health unit is supposed to look like. This also resulted in the students having a lack of privacy when caring for the patients and a lack of opportunities to learn from the appropriately trained registered nurses. A study conducted by Eweida et al. (2020) found that a psychiatric unit must be patient friendly and should be an environment that helps make patients more receptive to the treatment provided by staff. If the environment does not make the patient receptive to the staff’s treatment it may pose dangers because the patients may become aggressive and unresponsive to the care offered to them. In the study of Acı et al. (2022), some students reported the need to increase the number of psychiatric hospitals and to regulate the physical environment. Additionally, participants also suggested that the iron bars be removed, and the number of activity rooms be increased. The latter to be an environment wherein patients could spend time with their families on certain days, and also that the building be improved aesthetically.

Participants in this study reported that there was a lack of trained mental health nurses at the general hospital. This is an indication that few nurses are trained in mental health, hence the reason why the nursing students did not get to learn much from the nurses during their placement in the mental health unit at the hospital. On the contrary, they had to rely on their lecturers during clinical accompaniment to guide them where and when they struggled. These findings align with those of Rahmani, Mohammadi and Fallahi-Khoshknab (2021) who reported that the lack of the necessary professional knowledge for working in psychiatric wards is a challenge which leads to or causes the lack of interest of nurses and those who wants to work in psychiatric wards. In their study, Eweida et al. (2020) found that understaffing of registered psychiatric nurses in psychiatric units had a crippling effect on mental health of students which were related to the possibility of contracting coronavirus disease 2019 (COVID-19) infection. The general hospital is also affected by understaffing of psychiatric nurses and this has a ripple effect on student nurses because they are not properly guided when doing their practical at the hospital. In contrast, Rahmani et al. (2021) further reported that, fear over patient assault is also one of the leading causes of understaffing, stating that patients with psychiatric disorders are believed to be irritable and aggressive. Peters et al. (2015) assert that a lack of resources such as understaffing of psychiatric nurses leads to ineffective teaching and learning programmes for psychiatric nursing students and demotivation among nursing students. The current study found that nursing students faced the challenge of not completing their learning objectives within a short period of allocation. This short time frame for their mental health placement at the general hospital was a burning issue for the students because they were not exposed to many dilemmas or many opportunities to assess the patients with mental illness. These findings are similar to that of a systematic review conducted by Happell and Gaskin (2013) which revealed the attitudes of undergraduate nursing students towards mental health nursing. Findings from that study reported that students who receive more hours of theoretical preparation in mental health nursing and undertake longer clinical placements tend to develop a favourable attitude towards mental health compared to those who spent less time in mental health nursing during their placement. As previously discussed, the number of mentally ill patients in Namibia, but specifically in the region, contextual to this study, is on the rise. This brings about an urgent need to provide facilities within the region which can cater for the mentally ill patients, including outpatient and inpatient services. Furthermore, it is essential for nursing students in their undergraduate degree to develop an interest in mental health to further equip themselves with a post graduate diploma in mental health. This, however, is challenged by the absence of suitable services within the training hospital. In addition, the hospital in the northeast region of Namibia, although approved as a training hospital, lacks the specific facilities to cater for the placement of nursing students with requirements to meet within a comprehensive undergraduate curriculum.

### Strengths and limitations

The study provided a broader understanding and insight regarding the experiences of nursing students who were allocated for mental health clinical placement at the general hospital. The use of a qualitative, exploratory, descriptive and contextual design enabled the participants to narrate and interpret their experiences freely and also make suggestions for improvements. The study focused only on undergraduate fourth-year nursing students who were allocated to a general hospital in the north-east of Namibia for their mental health clinical placement. As such, it is not possible to generalise the findings from this study to any other undergraduate students who were allocated to other hospitals, as their contexts may differ, particularly to the availability of a specialised mental health facility.

## Conclusion

The use of a general hospital for clinical placements within an undergraduate nurse training course led to some concerns regarding the relevance and appropriateness of such experience within a nursing programme. Positive practice placement experience, associated with successful development of clinical skills, strongly influences future career choices in mental health and it is seen to have a significant impact on students’ attitudes towards mental health as an area of specialisation. Findings from this study can be used by the clinical nursing education office and faculty management team to improve the clinical training for undergraduate nursing students with respect to mental health. This study’s findings can be used to understand and evaluate whether the mental health clinical learning outcomes are being met or not and to make necessary changes to clinical placement strategies. As consumers of the training opportunities at health facilities, motivation towards the improvement of mental health facilities is something that needs to be driven from the training institution. However, the implementation thereof solely depends on the service provider, in this case, the Ministry of Health and Social Services in Namibia.
